# Constitutional Causes of Caries

**Published:** 1862-05

**Authors:** H. H. Black


### CONSTITUTIONAL CAUSES OF CARIES.

BY H. H. BLACK.

THERE is no subject at present claiming the attention of the Dentist, more neglected than the subject of caries induced by constitutional causes. This subject appears to be overlooked by many of the leading members of the profession, and subjects more directly conducive to pecuniary results allowed to occupy the field of their investigations. To judge from the multitude of notices that daily appear in our journals, of "great improvements in mechanical dentistry," the terms of "office rights," &c., the natural conclusion is that the great *desideratum* is *that* object most suitable for attaching letters patent. By this it is not to be understood that I oppose mechanical improvements, or *even dental patents*, if they are worth their weight in blank paper. But it is claimed that the over-anxiety in procuring letters patent, by too many of our profession, has been the means of diminishing, to a great extent, that investigation best calculated to benefit mankind.

That there is undoubtedly a large amount of caries induced by constitutional causes transmitted from parent to child, no one will contradict; and this caries I believe to first proceed from an unnatural and artificial mode of living which will require the co-operative skill of the dentist and physician, with the most vigilant care of parents, for several successive generations, to entirely remove. And a beginning in the right direction can not be made too soon; the community can not become too well or too soon informed on this subject. This beginning must be made by the dentist imparting such information to his patients as to enable them to fully understand the subject. His constant aim should be to convince them that caries is *not* a natural consequence, but the result of neglect and uncleanliness; and that the teeth of every individual will, by constant attention and proper treatment, continue in perfect health. The community must also become convinced of the large amount of caries arising from our unnatural mode of living, and the necessity of avoiding its consequences. The luxuries of civilized life would be hard to part with, and yet, by adhering to them, we bequeath imbecility upon generations to come. By consulting the writings of travelers to all barbarous nations on the globe, where man is living in a state of nature, deprived of the injurious luxuries of civilized life, whether it be under the equinoxial sun or amid ice-bound cliffs of the frigid zone, *their teeth are not known to decay.*

Any dentist who will carefully examine the physiology and pathology of the teeth, and also observe the fact of their not being liable to decay in men living in a state of nature, must be convinced that their decay is an unnecessary consequence, caused by man's neglect, and his artificial and unnatural mode of living.

What nation on the globe, it could be asked, have better teeth than the Brahmins of Hindoostan? And their preserving their teeth to extreme old age, free from all decay and tartar, is attributed to this deprivation of the luxuries of civilized life, and their constant, unremitting cleanliness of these organs; for it is a part of their religious ceremony—which is taught them as a holy duty in infancy—to rub their teeth for one hour every morning with the twigs of a fig tree.

Dr. Parmly says: "After more than thirty years' experience in dental surgery and minute observation, I am confident that the teeth of every individual may, by proper and daily friction, continue in perfect health to extreme old age."

In conclusion I will add, that in the summer of 1859 crossed the American continent. My route led me through the most populous tribes of the North Amrican Indians, which afforded frequent opportunities of examining and ascertaining the condition of the natives' teeth. Among the most filthy tribes, I occasionally observed small depositions of hard tartar on the inferior incisor teeth, with now and then a case of irregularity; but in no case during my whole travels did I find a single carious tooth until after I had approached those tribes bordering on the outskirts of civilization, that inhabit the western slopes of the Rocky Mountains, and who have been enjoying, to some extent, the luxuries of civilized life.

Truthfully it has been said, "were that part of the superstition of the Hindoos, which requires perfect cleanliness of the teeth, blended with the paternal instructions of this Christian land, how much more would be extended the sum total of human happiness! how many millions of human beings would be saved from days of excruciating agony! and how many thousands would be saved from sinking prematurely to the grave!"

